# Valvular heart disease: shifting the focus to the myocardium

**DOI:** 10.1093/eurheartj/ehac504

**Published:** 2022-09-28

**Authors:** Nina Ajmone Marsan, Victoria Delgado, Dipan J Shah, Patricia Pellikka, Jeroen J Bax, Thomas Treibel, João L Cavalcante

**Affiliations:** Department of Cardiology, Leiden Univesity Medical Center, Albinusdreef 2, 2300 RC, Leiden, The Netherlands; Department of Cardiology, Leiden Univesity Medical Center, Albinusdreef 2, 2300 RC, Leiden, The Netherlands; Department of Cardiology, Germans Trias i Pujol Hospital, Carretera de Canyet s/n. 08916 Badalona, Barcelona, Spain; Division of Cardiovascular Imaging, Weill Cornell Medical College, Houston Methodist DeBakey Heart & Vascular Center, 6565 Fannin St, Houston, TX 77030, USA; Department of Cardiovascular Medicine, Mayo Clinic, 200 First St. SW, Rochester, MN 55905, USA; Department of Cardiology, Leiden Univesity Medical Center, Albinusdreef 2, 2300 RC, Leiden, The Netherlands; Department of Cardiology, Barts Heart Centre and University College London, West Smithfield, London EC1A 7BE, UK; Department of Cardiology, Minneapolis Heart Institute at Abbott Northwestern Hospital, 800 E 28th St, Minneapolis, MN 55407, USA

**Keywords:** Valular heart disease, Mitral regurgitation, Aortic stenosis, Myocardial function, Myocardial fibrosis, Echocardiography, Magnetic resonance imaging

## Abstract

Adverse cardiac remodelling is the main determinant of patient prognosis in degenerative valvular heart disease (VHD). However, to give an indication for valvular intervention, current guidelines include parameters of cardiac chamber dilatation or function which are subject to variability, do not directly reflect myocardial structural changes, and, more importantly, seem to be not sensitive enough in depicting early signs of myocardial dysfunction before irreversible myocardial damage has occurred. To avoid irreversible myocardial dysfunction, novel biomarkers are advocated to help refining indications for intervention and risk stratification. Advanced echocardiographic modalities, including strain analysis, and magnetic resonance imaging have shown to be promising in providing new tools to depict the important switch from adaptive to maladaptive myocardial changes in response to severe VHD. This review, therefore, summarizes the current available evidence on the role of these new imaging biomarkers in degenerative VHD, aiming at shifting the clinical perspective from a valve-centred to a myocardium-focused approach for patient management and therapeutic decision-making.

## Graphical Abstract

**Graphical Abstract:**
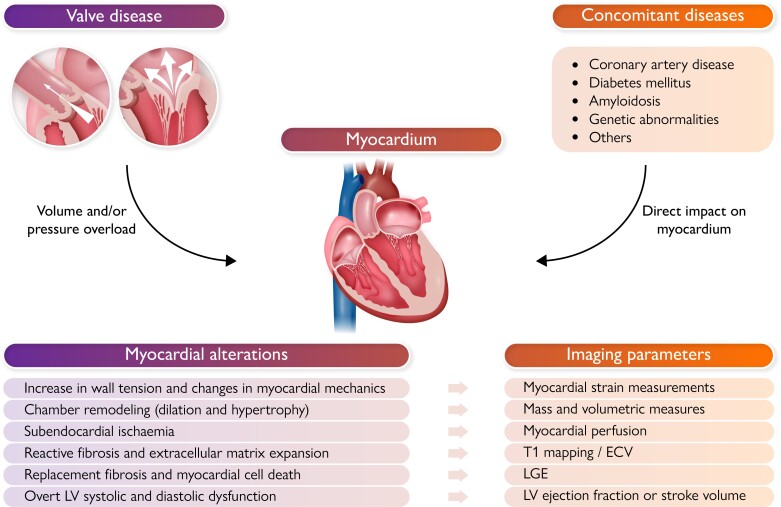
In the presence of severe valvular heart disease (and associated comorbidities), cardiac chambers are exposed to either pressure (as in aortic stenosis) or volume overload (as in mitral regurgitation), which triggers at first a series of adaptive mechanisms to release wall tension and maintain cardiac output (namely chamber hypertrophy and/or dilatation). However, these compensatory phenomena are soon followed by maladaptive myocardial changes such as reactive fibrosis, microvascular ischemia and cell death with replacement fibrosis, which substantially impair their function. New imaging biomarkers (namely obtained from echocardiography and cardiac magnetic resonance imaging) are capable of depicting the occurrence of such maladaptive changes with important potential impact on patient risk stratification and decision making. Particularly, global longitudinal strain has shown to be a sensitive marker of systolic dysfunction and cardiac magnetic resonance imaging has the key strength of myocardial tissue characterization, including fibrosis an inflammation, but also myocardial perfusion and energetics, and therefore offers an in-vivo “virtual histology”.

## Introduction

Valvular heart disease (VHD) is a major cause of morbidity and mortality worldwide, affecting >2% of the general population.^1^ While rheumatic disease remains the leading cause in low-to-middle income countries, in developed countries degenerative VHD, and particularly aortic stenosis (AS) and mitral regurgitation (MR) are the most prevalent, with an increasing frequency by an aging population and with an inherent high burden of comorbidities.^2^ Since no effective medical therapy is currently available, treatment of degenerative VHD relies on either surgical or transcatheter interventions. However, optimal timing of intervention remains a clinical challenge, balancing the risks of intervening too early, and therefore exposing patients to unnecessary peri-procedural and long-term complications, in contrast to a watchful waiting strategy, with the possible occurrence of sudden cardiac death or heart failure. Current guidelines^3,4^ recommend intervention based on VHD severity, presence of symptoms, and/or of left ventricular (LV) remodelling or reduced ejection fraction (EF). Despite the profound clinical implications, assessment of symptoms can be challenging: symptoms can be under-reported by the patient, could be related to concomitant comorbidities (pulmonary disease, coronary artery disease, atrial fibrillation, obesity) and, most importantly, they often appear at a late stage, when systemic and pulmonary circulation are significantly impaired and possibly irreversible myocardial damage has already occurred. When VHD is significant, cardiac chambers are exposed to either pressure (as in AS) or volume overload (as in MR), which triggers at first a series of adaptive mechanisms to release wall tension and maintain cardiac output (namely, chamber hypertrophy and/or dilatation). However, these compensatory phenomena are soon followed by maladaptive myocardial changes such as reactive fibrosis, microvascular ischaemia and cell death with replacement fibrosis, which substantially impair LV function, but only later result in a reduced left ventricular ejection fraction (LVEF). An impaired LVEF is therefore a strong prognosticator but, reflecting the complete exhaustion of these compensatory mechanisms, is a late marker of myocardial dysfunction, lacking sensitivity to depict the myocardial damages which already took place. To avoid the occurrence of irreversible myocardial damage, new biomarkers capable of depicting the occurrence of such maladaptive changes are advocated with important potential impact on patient risk stratification and on interventions’ results. To answer this need, advanced imaging may play a crucial role in identifying adverse myocardial remodelling at an early stage. Particularly, echocardiography is the first-line diagnostic modality, and along with strain imaging can provide an advanced assessment of myocardial mechanics; LV global longitudinal strain (LVGLS) has shown to be a sensitive marker of systolic dysfunction in various cardiovascular diseases including degenerative VHD.^5^ Cardiac magnetic resonance imaging (CMR) has the key strength of myocardial tissue characterization, including fibrosis and inflammation, but also myocardial perfusion and energetics, and therefore offers an *in vivo* ‘virtual histology’ of great value also in degenerative VHD.

The current review, therefore, summarizes the evidence on the role of imaging biomarkers of myocardial response in degenerative VHD (*Table 1*), and seeks to highlight their potential in shifting the focus of the clinical assessment from the valve to the myocardium, in order to improve patient management and therapeutic decision-making.

**Table 1:** Standard and novel myocardial imaging biomarkers that showed diagnostic and prognostic value in the management of patients with degenerative valvular heart disease

	Myocardial imaging biomarker	
	Echocardiography	CMR
**Primary mitral regurgitation**		
Standard	LVEDD LVEF LA diameter LA volume PAPs RV dimension and function (TAPSE, FAC)	LVEDD LV volumes and EF LV hypertrophy/mass RV volumes and function
New	LV GLS LV mechanical dispersion LA reservoir strain 3D LV volumes	LGE (replacement fibrosis) Extent Location ECV (interstitial fibrosis) GLS
**Aortic stenosis**		
Standard	LVEF LV dilatation LV hypertrophy Stress echocardiography	LVEF LV mass/hypertrophy
New	LV GLS LV myocardial work indices	LGE (replacement fibrosis) ECV (interstitial fibrosis) Amyloidosis assessment (including nuclear imaging) GLS T2 Perfusion for detection of epicardial coronary artery and/or microvascular disease
**Aortic regurgitation**		
Standard	LVEDD and LVESD LVEF Stress echocardiography	LV volumes and EF LV hypertrophy/mass LV diameters
New	LV GLS LV myocardial work indices 3D LV volumes	LGE ECV GLS
**Tricuspid regurgitation**		
Standard	RV dimension/area TAPSE FAC	RV volumes and EF RV mass RA area and volume
New	RV-PA coupling 3D RV volume and EF RV strain	LGE ECV RV strain

ECV, extracellular volume; EDD, end-diastolic diameter; EF, ejection fraction; FAC, fractional area change; GLS, global longitudinal strain; LA, left atrium; LGE, late gadolinium enhancement; LV, left ventricle; PAPs, systolic pulmonary artery pressure; RV, right ventricle; TAPSE, tricuspid annular plane systolic excursion.

## The myocardium in primary mitral regurgitation

MR is classified as primary when it is due to intrinsic lesions of the mitral valve, rather than to a disease of the LV as in secondary MR. Primary MR can be caused by congenital malformations or endocarditis, but most frequently is degenerative, either due to rheumatic disease or myxomatous infiltration as in mitral valve prolapse (MVP).^1,2^ MVP is the most common cause of MR in Western countries and two distinct phenotypes are generally recognized: fibro-elastic deficiency (FED) and Barlow’s disease.^6^ Although a clear distinction cannot always be made, patients with Barlow’s disease are usually younger and the mitral valve is characterized by thickened leaflets, with multi-segmental prolapse, chordal elongation, and typical annular abnormalities, such as dilatation, posterior disjunction, and abnormal displacement. In turn, patients with FED are typically older (and therefore with more comorbidities) and show thin or normal leaflets, with single segment prolapse/flail by chordal rupture. Regardless of the aetiology, when severe MR develops, left atrial (LA) and LV dilatation occurs as adaptive mechanisms to accommodate the volume overload and maintain cardiac output. In most cases, during this phase, pulmonary pressures remain within normal limits, LVEF remains falsely normal or supra-normal (considering the reduced afterload) and patients are asymptomatic. However, a chronic increase in LV wall stress leads to non-ischaemic fibrosis and adverse myocardial remodelling, which may progress to overt LV dysfunction. Also, the further upstream effect of a severe MR will eventually lead to elevated pulmonary pressures, which often induce right ventricular (RV) dilatation, secondary tricuspid regurgitation (TR), and ultimately RV dysfunction. However, this pathophysiological cascade is not homogeneous in all patients, and the mechanisms by which the cardiac chamber remodels to hemodynamically adapt to severe MR can vary significantly. Age, mechanism of MR, and associated comorbidities may influence cardiac remodelling response to volume overload. In Barlow’s disease particularly, a specific cardiomyopathy has been suggested as part of the phenotype leading to a LA and LV dilatation disproportionate to the severity of MR.^7^ Current guidelines, beyond symptoms, consider LVEF and end-systolic diameter as main gatekeepers (Class I) to surgery; additional reasons for intervention (Class II) are also considered atrial fibrillation, LA dilatation and increased pulmonary pressure.^3,4^ However, LVEF may remain normal for relative long time without properly reflecting ongoing important damages of the myocardium; also, the cut-off values of the other echocardiographic parameters of chamber remodelling have been derived mainly from symptomatic patients,^8^ when myocardial structural changes may have already occurred and may not be reversed after intervention. Therefore, there is still a need for new imaging biomarkers able to better understand myocardial involvement in degenerative MR, possibly at an early stage, and to refine indication for intervention.

### Echocardiographic markers of adverse cardiac remodelling

**Figure 1:**
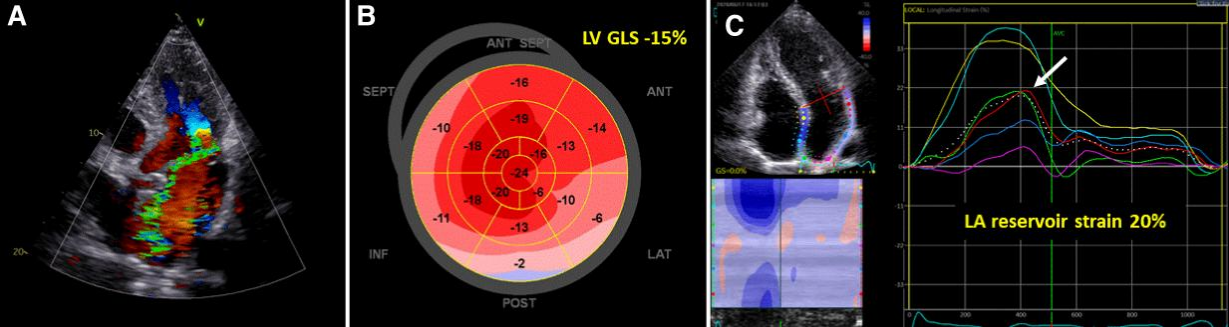
(*A*) Assessment of left ventricular global longitudinal strain and left atrial reservoir strain by speckle tracking echocardiography, in a patient with mitral valve prolapse and severe mitral regurgitation. (*B*) A bull’s eye plot displays the regional longitudinal strain for each left ventricular segment with a colour code; in this patient, global longitudinal strain (average of the 17 segments) was significantly impaired (being left ventricular global longitudinal strain value considered normal between −18 and –20%). (*C*) The longitudinal strain curve of the left atrial derived from the apical 4 chamber view (dotted line as average of the segments), and the peak positive strain is the left atrial reservoir strain, which was also impaired in this patient (as <22%).

Standard echocardiography can provide all the parameters currently used to recommend intervention in degenerative MR, including LV diameter and EF, LA diameter and volume, and pulmonary pressure. Particularly, the presence of pulmonary hypertension and also of RV dysfunction (possibly signs of exhaustion of the LV and LA compensatory mechanisms) has been associated with poor outcomes, and patients presenting with these characteristics should undoubtedly be referred to surgery at an earlier stage.^9^ However, also waiting for LV and LA dilatation may increase the risk of incipient myocardial damage that is not detectable by conventional echocardiographic techniques. Strain echocardiography has shown that in patients with severe primary MR and a mean LVEF >60%, LVGLS was significantly impaired as compared to controls, and decreased with increasing LV diameters and regurgitant volume.^10^ Furthermore, the prognostic value of LVGLS to predict post-operative LV dysfunction and mortality after surgery in patients with severe primary MR has been demonstrated in several studies. Among 593 patients with severe primary MR, those who presented an LVGLS ≥20.6% had better outcomes after surgical mitral valve repair as compared to those patients with an LVGLS <20.6% (*Figure 1*).^11^ Similar results were demonstrated also in asymptomatic patients with severe primary MR.^12,13^ Of note, the relatively preserved values of LVGLS could be related to the reduced afterload since the LV empties into a low chamber pressure, the LA.

Based on strain imaging, the time delay of LV longitudinal strain can be derived as a measure of LV mechanical dispersion which has been associated with the occurrence of ventricular arrhythmias. In 610 patients with MVP, those with symptomatic ventricular arrhythmias had worse LVGLS and more pronounced mechanical dispersion as compared to patients without ventricular arrhythmias, despite having similar LVEF;^14^ also significant mitral valve annular abnormalities were observed, including annular dilatation and disjunction, described in MVP patients as a separation between the LA wall at the level of the mitral valve junction with the LV free wall. These results highlight the presence of a cardiomyopathic process with structural abnormalities that affect myocardial mechanics at an earlier stage than LVEF.

Strain measures can also be applied to the LA to assess its reservoir function and by reflecting LA compliance, may therefore identify early signs of LA remodelling. In a recent study, LA reservoir strain was independently associated with all-cause mortality (with a proposed cut-off value of 22%) in patients undergoing mitral valve repair for severe primary MR, and with incremental prognostic value over current clinical and echocardiographic factors (*Figure 1*).^15^

### CMR markers of adverse cardiac remodelling

**Figure 2:**
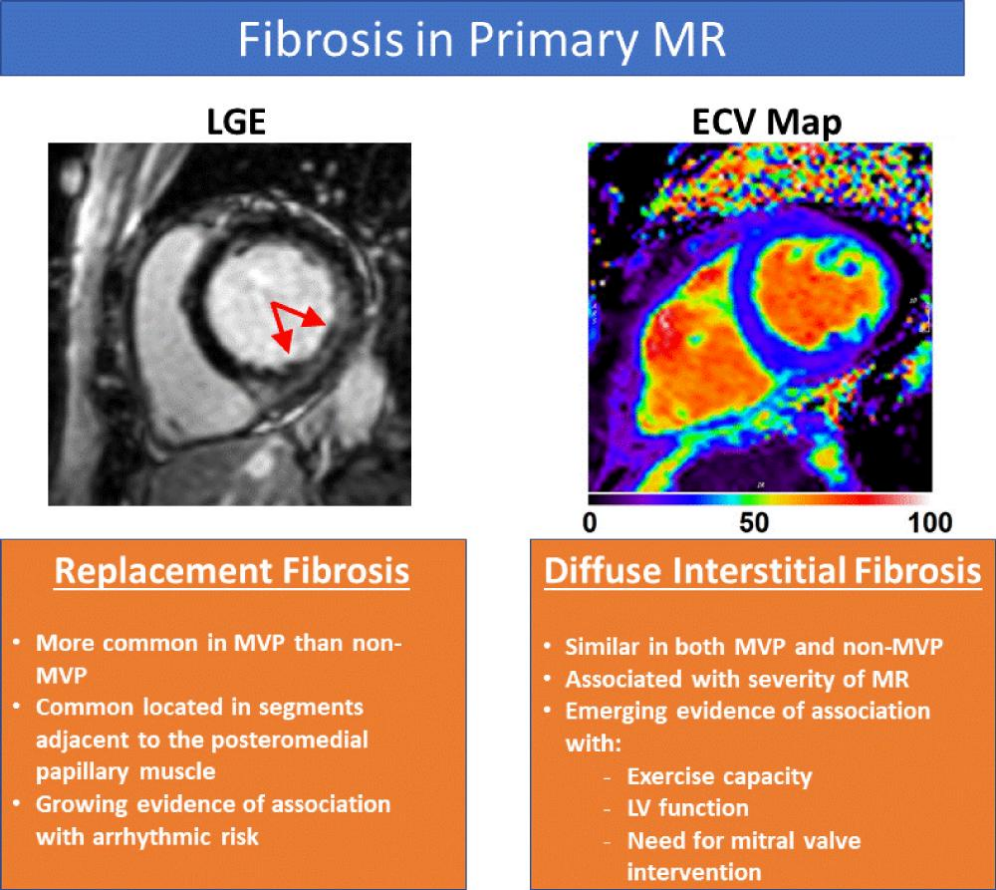
Assessment of myocardial replacement and interstitial fibrosis in patients with primary mitral regurgitation. ECV, extracellular volume; LGE, late gadolinium enhancement; LV, left ventricular; MVP, mitral valve prolapse.

Considering the key parameters to trigger MR intervention, CMR is a well-established method for quantifying LV dimension and EF, with high reproducibility and obviating the need for geometric assumptions. However, LV volumes may be a more reliable marker of LV remodelling and in a series of asymptomatic MR patients, CMR-derived LV end-systolic volume index demonstrated a higher predictive value than echo-derived LV end-systolic diameter for survival free of mitral surgery.^16^ However, the ability to assess myocardial composition is the unique feature of CMR which has provided novel markers of myocardial tissue remodeling in patients with primary MR (*Figure 2*).^17^ Replacement myocardial fibrosis as assessed by late gadolinium enhancement (LGE) has been noted in the setting of primary MR with several key caveats.^18^ First, LGE is much more prevalent in primary MR due to myxomatous MVP than other non-myxomatous aetiologies, supporting the hypothesis that these specific mitral valve alterations are also associated with myocardial abnormalities and a pro-fibrotic milieu. Second, LGE is commonly located in the segments adjacent to the posteromedial papillary muscle (e.g. inferolateral or inferior walls) and to the mitral valve, suggesting also a mechanical trigger (pulling of the elongated chorda and hyperdynamic annular motion and disjunction) to the development of fibrosis.^19^ Third, the prevalence of LGE increases with the severity of MR, being as high as 50% in patients with severe MR, and confirming the irreversible myocardial damages secondary to the volume overload.

Several recent studies have also demonstrated a prognostic implication of replacement fibrosis in the setting of MVP. Starting from autopsy studies, analysis of sudden cardiac death victims with MVP revealed the presence of LV replacement fibrosis and with the propensity of segments adjacent to the posteromedial papillary muscles.^19^ By using CMR in a US-based series, patients with MVP and evidence of LGE experienced a 7.7% rate of arrhythmic events (sudden cardiac death, aborted sudden cardiac arrest, sustained or inducible ventricular arrhythmia) as compared to 2.7% for MVP patients without replacement fibrosis, and 0.6% for the group with primary MR due to non-MVP aetiology (in which the prevalence of replacement fibrosis was very low).^18^ Similarly, a French study of MVP patients demonstrated a higher likelihood of arrhythmic events in presence of LGE.^20^ Furthermore, MVP patients with replacement fibrosis experienced a 2.6-fold higher rate of cardiac death, heart failure, new-onset atrial fibrillation, arterial embolism, or life-threatening ventricular arrhythmia, supporting the growing evidence that LGE is associated with an increased risk of adverse cardiovascular events in these patients.

CMR can also quantify myocardial extracellular volume (ECV) by T1 mapping, based on the change in T1 times before and following administration of gadolinium contrast.^17^ Studies have shown myocardial ECV to be correlated with histologically quantified diffuse interstitial myocardial fibrosis in several medical conditions,^21^ including VHD. A cross-sectional study of patients with asymptomatic moderate or severe primary MR noted that ECV was correlated with total exercise time, metabolic equivalent, and peak oxygen consumption.^22^ In a large study of patients with chronic primary MR, ECV was higher in patients with symptomatic MR.^23^ Furthermore, when followed longitudinally, patients with an ECV ≥30% experienced a higher likelihood of death or need for mitral valve surgery^24^ and a recent study also showed an association between ECV (particularly in the basal segments) and complex ventricular arrhythmias.^25^ While still an emerging marker, current evidence suggests that ECV may represent a marker of maladaptive remodelling and subclinical decompensation in chronic primary MR.

Recently, a prospective multicentre study enrolled 104 patients with primary MR who underwent CMR before and on an average of 8 months after mitral valve repair.^26^ After surgery, a significant reduction in ECV fraction and indexed ECV, proportional to their preoperative expansion was observed, but not in LGE. These findings suggest that interstitial reactive fibrosis is probably reversible while replacement fibrosis is not. Also, preoperative ECV predicted the degree of post-operative remodelling irrespective of symptoms, highlighting that although patients with LGE and interstitial fibrosis still benefit from surgery, they are less likely to demonstrate reverse remodelling after surgery and a deleterious effect on LV function remains.

Finally, CMR tagging and feature-tracking also allow for assessment of myocardial deformation (strain), and initial studies in patients with chronic primary MR have shown namely a decrease in regional circumferential strain, persistent or occurring *de novo* even after mitral valve repair.^27,28^

Further studies are needed to confirm whether these CMR measures can help in timing mitral valve interventions in these patients, and if mitral valve repair is a strategy to lessen the excess risk conferred by replacement and diffuse interstitial fibrosis.

## The myocardium in aortic stenosis

In patients with severe AS, LVEF is often preserved and LV hypertrophy develops gradually to reduce wall stress and maintain cardiac output.^29^ In parallel to the progressive LV hypertrophy, there is an increase in interstitial fibrosis and myocyte apoptosis, due to oxygen supply-demand mismatch and myocardial ischaemia. If the valve is left untreated, the LV myocardium develops areas of myocyte loss and three distinct patterns of fibrosis have been described: thickened endocardium, development of irreversible microscars particularly in the sub-endocardium with a gradient from the inner to the outer third of the myocardium, and inter-fiber and perivascular fibrosis throughout the myocardium.^30^ These important structural abnormalities lead at a later stage to LV systolic dysfunction, which when overt has been associated with poor outcome and included as an important criterion to indicate surgery in patients with severe AS with or without symptoms.^3,4^ Risk stratification and the best timing for prophylactic intervention, however, remain controversial in asymptomatic patients with preserved LVEF, and is currently being evaluated in several prospective randomized controlled trials. Other prognostic factors should be considered in patients with severe AS, as for example it has been demonstrated for the right heart involvement (pulmonary hypertension, TR, and RV dysfunction), shown to be associated with poor prognosis even in asymptomatic patients.^31,32^ In addition, transthyretin cardiac amyloidosis (ATTR) has been recognized as an important combined myocardial and aortic valve pathology, which poses diagnostic challenges due to presentation with low-flow, low-gradient AS and, more importantly, is characterized by a more severe phenotype with more frequently heart failure, arrhythmias, and higher mortality.^33–37^

### Echocardiographic markers of adverse cardiac remodelling

**Figure 3:**
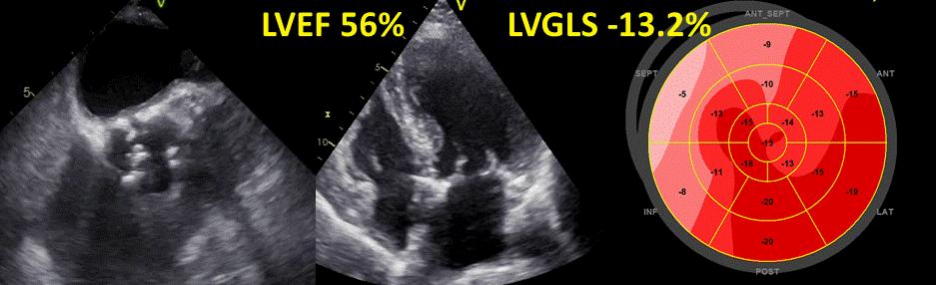
Echocardiographic assessment of a patient with severe aortic stenosis. Despite a preserved left ventricular ejection fraction, the value of left ventricular global longitudinal strain is significantly impaired. For description of the strain analysis, see also legend of *Figure 1*.

The functional abnormalities resulting from the above-mentioned structural changes are better detected by advanced echocardiographic parameters such as LVGLS, which are more sensitive than LVEF. Several studies showed that LVGLS is impaired in patients with severe AS even in case of preserved LVEF and absence of symptoms (*Figure 3*). In particular, Vollema *et al.*^38^ compared 220 asymptomatic patients with severe AS to 220 age- and sex-matched controls without AS; both groups had similar, preserved LVEF, but LVGLS was significantly reduced in the patients with severe AS (17.9 ± 2.5% vs. 19.6 ± 2.1%, *P* < 0.001). Dahl and coworkers^39^ published a systematic review that confirmed that patients with severe AS are often asymptomatic, present with normal LVEF (≥50%) but have an impaired LVGLS. Subsequent studies showed also the strong prognostic value of LVGLS in patients with severe AS.^40^ In the aforementioned study by Vollema and colleagues,^38^ in patients with asymptomatic severe AS and preserved LVEF, LVGLS deteriorated during a median follow-up of 12 months (from 18.0 ± 2.6% to 16.3 ± 2.8%, *P* < 0.001) when treated conservatively. Moreover, patients with reduced LVGLS at baseline were at higher risk of developing symptoms and need for aortic valve replacement (AVR) during follow-up. An individual participant data meta-analysis,^41^ totalling 1067 asymptomatic patients with severe AS and LVEF >50%, showed that a cut-off value of LVGLS <14.7% was associated with a 2.5-fold increased risk of death. When considering only patients with an LVEF ≥60%, the association between LVGLS and all-cause death remained significant.

The pressure overload characteristic of AS has an influence on the reduced value of LVGLS and therefore, after AVR, an improvement in LVGLS has been demonstrated.^42^ However, LVGLS may remain impaired after surgery in many patients suggesting that the structural changes have not been reversed fully or the changes are not only caused by LV hypertrophy. In patients with severe AS and ATTR amyloidosis, a thicker LV, more impaired LVGLS, lower stroke volume, and worse LV diastolic function were observed as compared to patients with severe AS only.^36^ Interestingly, the typical pattern on LVGLS with relative apical longitudinal strain sparing was observed with similar frequency in patients with and without ATTR amyloidosis.

LVGLS and echocardiography-derived LV systolic pressure (by adding the mean aortic valve gradient to the aortic systolic pressure) can be incorporated to construct pressure-strain loops of the LV and obtain myocardial work measures which take into account LV afterload: in a recent study, LV global work index and constructive work showed an independent association with heart failure symptoms in patients with severe AS.^43^

### Other imaging markers of adverse cardiac remodelling

**Figure 4:**
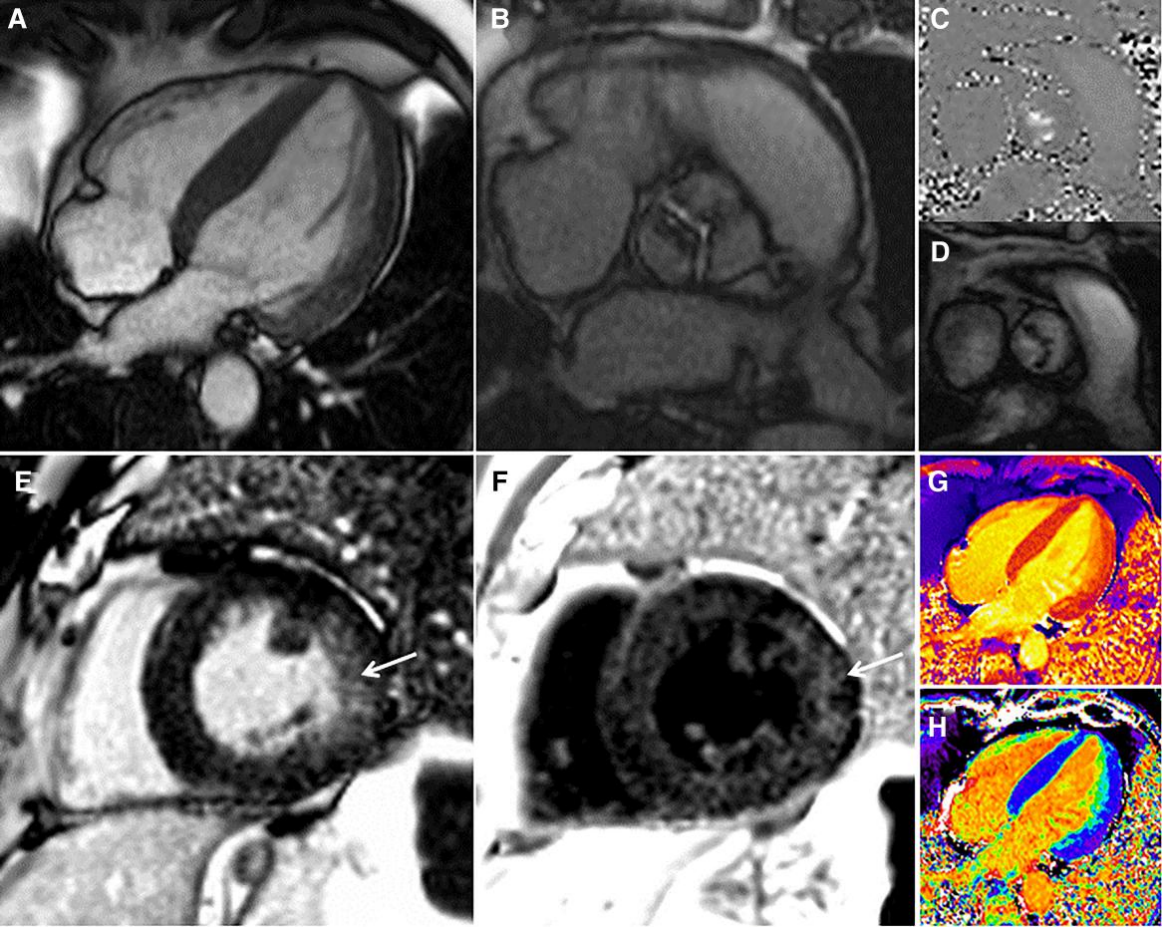
Assessment by magnetic resonance imaging of a patient with critical aortic stenosis. A 4-chamber bSSFP-cine image (*A*) showing normal left ventricular cavity size with concentric hypertrophy. Short-axis bSSFP-cine image (*B*) en-face view of the aortic valve demonstrating fusion of the left and right coronary cusp and a planimetered aortic valve area of 0.6 cm^2^. Phase-contrast imaging just above the aortic valve (*C* + *D*) demonstrating a peak velocity of nearly 5 m/s. Bright (*E*) and dark (*F*) blood late gadolinium enhancement images demonstrating patchy, non-infarct scar in the lateral wall. A native T1 map (*G*) and extracellular volume fraction map (*H*) demonstrate no evidence of myocardial infiltration (septal T1 1040 ms, lateral wall T1 1060 ms, normal <1060 ms; septal ECV 26%, lateral wall ECV 28%, normal <30%).

CMR is more sensitive than echocardiography to identify altered global LV geometry (radius and wall thickness, or mass volume ratio), and has revealed marked sex dimorphism in the remodelling response to severe AS. Men show higher indexed LV mass, lower LVEF, and increased myocardial stiffness, while women present more concentric remodelling with higher relative wall thickness and LVEF; however, the scale of the differences is being increasingly recognized with apparently more maladaptive myocardial response to AS in men.^44–47^ More importantly, CMR allows the visualization of both patchy non-infarct pattern scar by LGE imaging^48,49^ as well as the diffuse fibrosis in the mid-myocardium using T1 and ECV (*Figure 4*). With progressive AS, LGE accumulates over time, slowly in mild AS (with minimal annual change), but faster in moderate and severe AS with an apparent acceleration trajectory of scar extent.^50^ Prevalence of LGE in severe AS ranges from 27 to 51%, is associated with more severe valvular stenosis and worse LV systolic and diastolic function; furthermore, it correlates with histology and appears to be irreversible at 9 and 12 months after surgical AVR.^51–56^ Importantly, LGE independently predicted all-cause and cardiovascular mortality, regardless of type of LGE (infarct pattern vs. non-infarct pattern) or intervention (surgical or transcatheter).^57^ After AVR, *de novo* LGE may occur in between 5 and 18% of patients but myocardial vulnerability during surgery is not yet well understood.^57–61^ Whether early intervention guided by LGE improves survival is currently under investigation in the EVOLVED trial (NCT03094143), assessing early intervention in asymptomatic patients with LGE.^59^

Beyond LGE, T1 mapping and ECV quantification allow assessment of diffuse fibrosis that precedes irreversible focal fibrosis.^60^ Diffuse fibrosis prior to AVR has been shown to predict symptomatic and LV function improvement.^61–63^ In a recent multicentre study in 440 patients with severe AS prior to surgery, ECV fraction was independently associated with cardiovascular and all-cause mortality.^64^ Importantly, reverse remodelling after AVR was associated with early normalization in LV function within the first 6 months, and LV mass regression in the first 6 to 12 months with up 20–30% LV mass reduction at 1 year.^65–73^ Of note, early mass regression was greater in the presence of more LV hypertrophy, and in absence of scar.^74^

ECV quantification allows further interrogation of this process by splitting LV mass into a matrix and cell compartment. Prevalence of increased myocardial ECV in severe AS (using the threshold of >28%) ranges from 33% up to 54% depending on the studied population.^56,64^ Early ECV data interrogating LV mass regression at 6 months post-AVR noted cellular regression without significant extracellular matrix changes,^75^ but more recent data demonstrated both cellular and matrix regression at 1 year (with the cellular response greater than the interstitial matrix response); scar by LGE however was irreversible.^56^ This highlights that myocardial compartments are plastic, providing scar is absent.

LV myocardial strain as measured by CMR was also shown in initial studies on AS, to discriminate between asymptomatic vs. symptomatic patients and to be associated with outcomes after surgical or transcatheter interventions.^27,76^

Nuclear scintigraphy has not played a significant role in the management of AS until recently, when bone scintigraphy (tracer is DPD, HMD, or PYP depending on the country) has been increasingly used to diagnose ATTR amyloidosis. Other imaging modalities, including CMR but also ECV by computed tomography can also help in characterizing myocardial involvement in this disease: the AS-amyloid prevalence in severe AS patients ranges between 8 and 16% depending on the age of the patients but in patients above the age of 75 years appears to affect 1 in 8.^33–37^ In principle, AVR should not be withheld from patients with dual pathology but patients should be considered for amyloid-specific therapies.^77^

## The myocardium in aortic regurgitation

Acute aortic regurgitation (AR) most often results from endocarditis, aortic dissection, chest trauma, or iatrogenic injury. When severe, acute LV volume overload generally leads to a low output state and pulmonary oedema. Thus, early surgical intervention occurs prior to the hemodynamic myocardial consequences which are expected in chronic AR. In contrast, in chronic severe AR, the LV faces a prolonged volume overload and relative pressure overload, with adaptive eccentric hypertrophy with cardiomyocyte growth by addition of new sarcomeres in series, and therefore increase in LV volumes and mass (both cellular and interstitial).^78^ LV dilatation is considered an important criterion in current guidelines for the management of patients with severe AR by using LV linear dimensions.^3,4^ However, it is well known that LV linear dimensions have limitations and volumetric measurements by echocardiography, especially when with three-dimensional imaging,^79^ can more precisely and accurately identify the complex LV remodelling which occurs in chronic AR.^80,81^ Echocardiographic LV volume assessment has been shown to have good reproducibility in patients with moderate-severe and severe AR and a LV end-systolic volume ≥45 mL/m² was independently associated with all-cause mortality as well as cardiac symptoms.^80^

The chronic volume (and pressure) overload which characterizes AR leads also to the development of myocardial fibrosis, diastolic dysfunction and ultimately impairment of contractile function.^78^ In a population of 41 patients with ≥ moderate-severe chronic AR, advanced diastolic dysfunction including pseudo-normalization or restrictive filling was present in 25% and has been associated with worse outcome after AVR.^82^

Finally, mortality in chronic AR increases by reduced LVEF, which however occurs at a late stage in the natural history of this VHD, and several studies have suggested that valve intervention should be considered at an earlier stage of ventricular remodelling than recommended by guidelines.^83–85^ In this regard, the functional consequences of the maladaptive processes to volume overload might be better reflected by myocardial strain measurements. Patients with severe AR and LVEF >50% have shown impaired values of LV longitudinal, circumferential and radial strain by echocardiography as compared to controls.^86^ When dividing patients according to the presence of symptoms, symptomatic patients had also more impaired LV longitudinal (14.9 ± 3.0 vs. 16.8 ± 2.5%, *P* < 0.001), circumferential (17.5 ± 2.9% vs. 19.3 ± 2.8%, *P* = 0.001), and radial (35.7 ± 12.2% vs. 43.1 ± 14.7%, *P* = 0.004) strains as compared to asymptomatic patients. Furthermore, LVGLS has been associated with all-cause death in 865 patients with severe AR who were asymptomatic or mildly symptomatic and had an LVEF ≥50%. Alashi *et al.*^87^ showed, that patients with an LVGLS <19.5% had higher mortality rates at 6 years follow-up as compared to patients with an LVGLS ≥19.5% (17% vs. 11%, *P* < 0.01). Each 1% absolute worsening in LVGLS was independently associated with all-cause death with a hazard ratio of 1.11.

To integrate the loading conditions in the assessment of LV systolic function, the calculation of pressure-strain loops and myocardial work has been proposed also in patients with chronic severe AR. Meucci *et al.*^88^ showed in 57 patients with moderate and severe chronic AR and preserved LVEF who were referred for surgical AVR, that the majority of the patients had normal or increased myocardial work. However, after aortic valve surgery, 28% of patients presented impaired LV myocardial work. The post-operative impairment of LV global myocardial work was associated with a lesser degree of LV reverse remodelling suggesting that there was prior to surgery a more advanced LV myocardial remodelling.

**Figure 5:**
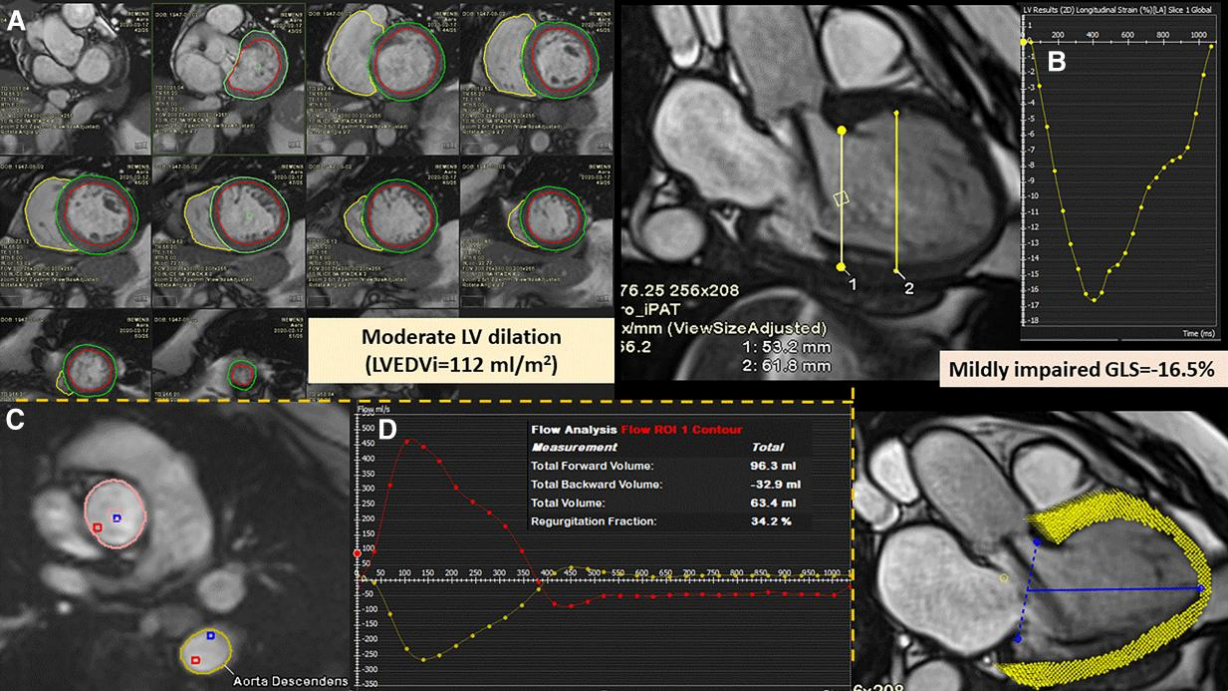
Magnetic resonance imaging for the assessment of aortic regurgitation severity and left ventricular remodelling (dilatation and dysfunction). A 72-year-old asymptomatic female patient with trileaflet aortic valve and echocardiogram showing moderate aortic regurgitation. (*A*) Breath-held short-axis stack cine acquisition for calculation of left ventricular volumes, ejection fraction, stroke volume, and left ventricular mass. There is moderate left ventricular dilatation (LVEDVi 112 mL/m^2^) despite a basal LVEDd measurement of 5.3 cm, vs. mid-left ventricular cavity (6.2 cm) correlates better with the volumetric mild left ventricular dilatation; left ventricular mass, wall thickness and LVEF (62%) are preserved. However in (*B*), global longitudinal strain is impaired = −16.5% (see the strain curve displayed in the upper panel and the myocardial overlay of the feature-tracking post-processing analysis in the lower panel). (*C*) 2D phase-contrast through-plane acquisition at the level of the ST junction for forward and backward flow and volume calculation. (*D*) Red curve indicates ascending aorta flow, whereas yellow curve, descending thoracic aorta. Severe aortic regurgitant fraction (34%) is identified. Holodiastolic retrograde flow in the descending thoracic aorta (yellow curve above the baseline) is a specific and supportive finding. Mild mitral regurgitation also identified (regurgitant volume 19 mL; regurgitant fraction 17%).

**Figure 6:**
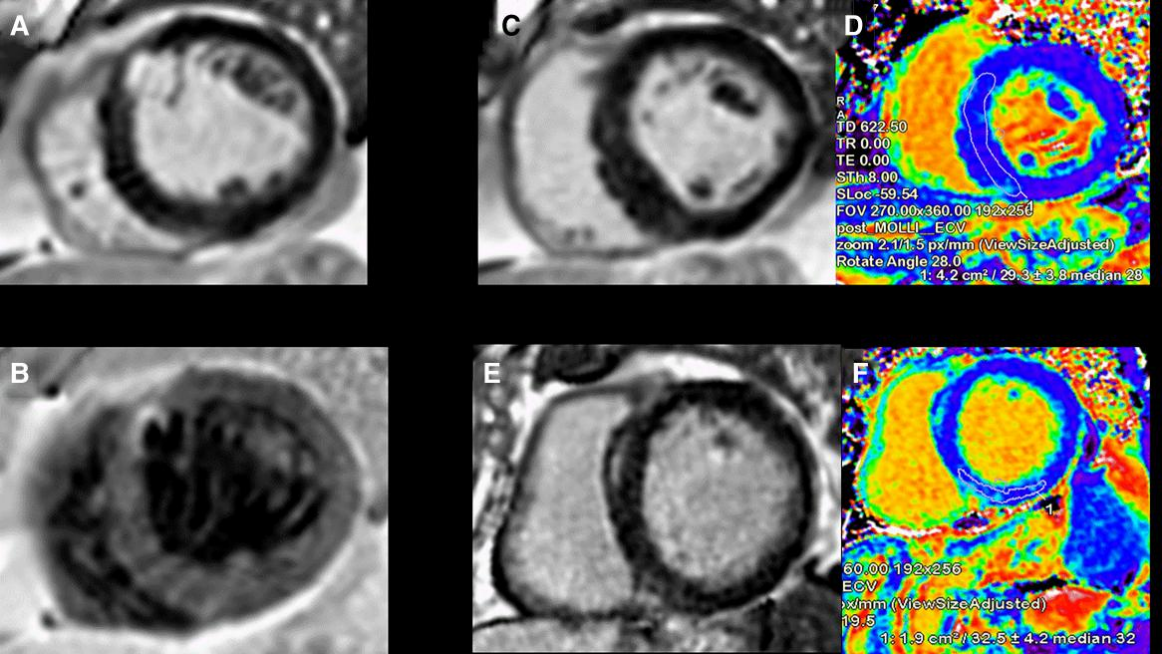
Patterns of myocardial fibrosis seen by magnetic resonance imaging in patients with chronic aortic regurgitation. (*A*) and (*B*) Discrete subendocardial infarction at mid anteroseptum; (*C*) and (*D*) increased wall thickness, but normal left ventricular mass, and no replacement myocardial fibrosis. Extracellular volume fraction (28%) is upper normal; (*E*) and (*F*) increased wall thickness with non-ischaemic midwall fibrosis at the basal anteroseptum and elevated extracellular volume fraction (32%), supportive more advanced left ventricular remodelling.

CMR evaluation may provide incremental value over echocardiography,^89^ namely by a better characterization of LV remodelling, precise quantification of AR severity, but more importantly identifying the development of myocardial fibrosis (*Figures 5 and 6*). Presence of either ischaemic or non-ischaemic myocardial fibrosis identified by CMR was seen in up to a third of patients chronic AR.^90^ Importantly, it was associated with 2.5-fold increase in mortality and mitigated by AVR. Particularly, ECV fraction was shown to have a strong correlation with AR severity measured by aortic regurgitant fraction, and both were associated with worse outcomes in chronic AR patients.^91^

When feature-tracking CMR was used to measure strain, LVGLS showed to be impaired early in the course of the disease and a marker of AR severity, while circumferential and radial strain worsened later and were associated with outcome, namely the need for aortic valve surgery.^92^

In summary, given the current widespread use of volumetric measurements and improved standardization, future guidelines should support the use of volumetric rather than linear measurements of the LV, either obtained by echocardiography or by CMR. Assessment of myocardial strain and fibrosis appears very promising for further risk stratification in severe chronic AR.

## The myocardium in tricuspid regurgitation

The aetiology of TR can be quite heterogeneous due to the interplay between the right atrium (RA), the RV, tricuspid annulus and pulmonary pressures, but also to leaflet morphology and other contributors such as atrial fibrillation and presence of intracardiac devices.^93^ The type and extent of RA and RV remodelling induced by TR is crucial for the management of these patients, and has been included as last topic of current review to create further awareness on the importance of standard and new imaging biomarkers to assess myocardial involvement of the right heart.^93^ The myocardial structural changes, including accumulation of collagen in the extracellular matrix, may differ significantly across the spectrum of TR aetiologies and severity.^94^ The RV histological changes are more pronounced in pressure overload conditions as compared to volume overload circumstances, and increased myocardial fibrosis has been described in experimental and clinical models particularly as results of pulmonary hypertension.^95–97^ However, combined pressure and volume overload is common in these patients whose overall myocardial remodelling and insult are amplified.^95,97^ Pressure-volume overload may lead also to RV ischaemia which further enhances myocardial fibrosis development. These structural changes all lead ultimately to impaired RV functional parameters which underlies the poor outcomes of these patients. It is therefore important to identify sensitive functional and anatomical imaging biomarkers to better risk stratify patients with TR. However, most of current functional parameters are influenced by the loading conditions (preload and afterload), presence of ischaemia, pericardial constraint and the interventricular dependence. Therefore, any RV functional imaging is an imperfect indirect marker of RV structural remodelling (myocardial fibrosis).

Bearing in mind those limitations, echocardiography is the imaging technique most frequently used in the evaluation of the patients with severe TR. Among the various parameters of RV function, longitudinal strain has demonstrated incremental prognostic value over RV fractional area change and tricuspid annulus plane systolic excursion (TAPSE). Among 896 patients with moderate and severe TR, RV longitudinal strain detected more frequently RV dysfunction as compared to RV fractional area change or TAPSE, and each 1% impairment in RV longitudinal strain was independently associated with increased all-cause death (hazard ratio 1.029, *P* = 0.003).^98^ To account for the afterload, especially important among patients with pulmonary hypertension and secondary severe TR, Fortuni and coworkers^99^ showed that the ratio between TAPSE and systolic pulmonary artery pressure reflected the RV to pulmonary arterial (RV-PA) coupling and had important prognostic implications. Patients with RV-PA uncoupling (defined by a ratio between TAPSE and systolic pulmonary arterial pressure <0.31 mm/mmHg) had 46% increased risk of all-cause death as compared to patients with preserved RV-PA coupling.

**Figure 7:**
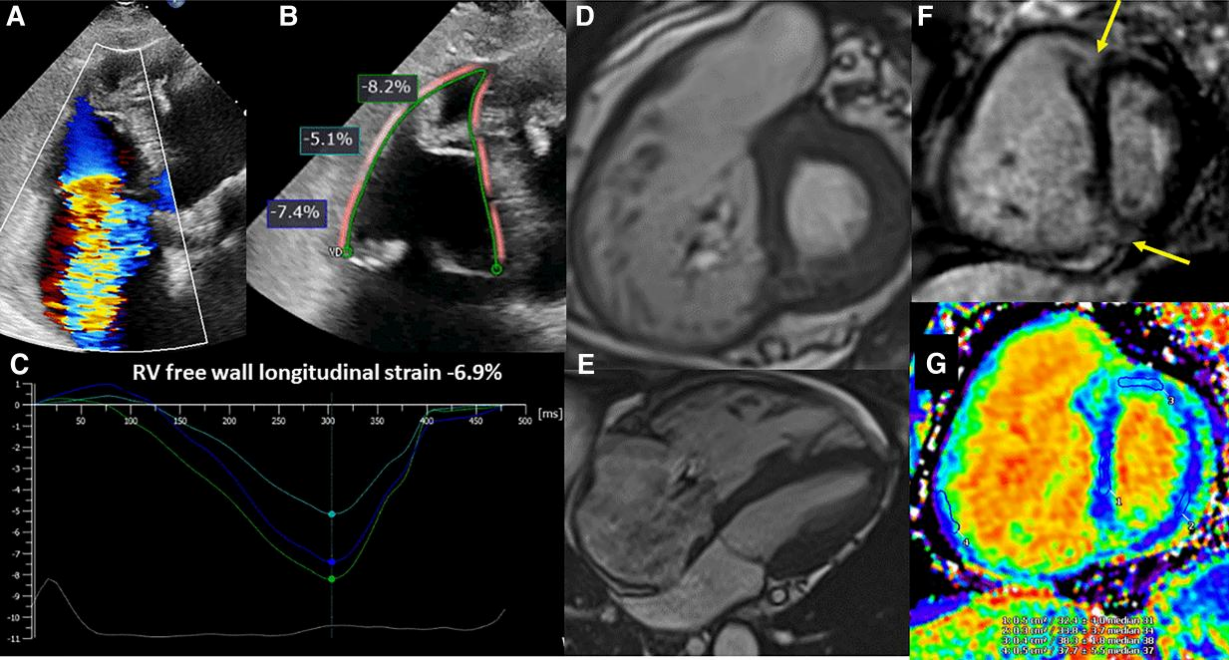
Assessment of right ventricular dysfunction and fibrosis in severe tricuspid regurgitation. (*A*) An example of a patient with massive tricuspid regurgitation and dilation of the right ventricle. In (*B*), the region of interest to measure right ventricular longitudinal strain with speckle tracking echocardiography is shown and the regional values of longitudinal strain in the free wall are presented. The time to peak longitudinal strain of the basal, mid, and apical segments of the lateral wall are presented in (*C*). (*D*) and (*E*) show the short-axis of the right ventricular at the level of the tricuspid valve and the 4-chamber view respectively obtained with cine-cardiac magnetic resonance imaging . On late gadolinium enhancement cardiac magnetic resonance imaging sequences, the macroscopic fibrosis can be observed in the junctional areas between the right and the left ventricles (*F*, arrows). Using T1 mapping techniques, microscopic fibrosis of the right and left ventricles can be measured (*G*).

Difficulties for CMR performance in this population are related to common presence of atrial fibrillation and intracardiac devices which can create artefacts. Nonetheless, the growing use of CMR in TR patients has led to a better understanding of its importance for this patient population for the following two main reasons: (i) being the gold standard for quantification of RV remodelling and RV function, uncovering the shortcomings of 2D echocardiography, and (ii) it evaluates using LGE the extent and pattern of myocardial fibrosis leading to the diagnosis of the underlying cardiomyopathy^100^ (*Figure 7*).

Ongoing transcatheter tricuspid replacement trials use cardiac computed tomography for anatomical screening and procedural planning but have not yet leveraged the full analytical capabilities of this imaging modality such as its functional assessment, quantification of TR severity (via measurement of effective regurgitant orifice area), myocardial strain or myocardial ECV fraction, a surrogate of diffuse myocardial fibrosis.^101^

## Clinical translation, implementation, and conclusions

In degenerative VHD, although valve stenosis or regurgitation are the primary insult, is the myocardial response that determines how the insult is tolerated and the prognosis of the patients, and should therefore drive the decision on in whom and when to intervene. Novel imaging biomarkers are therefore advocated to better understand myocardial changes secondary to volume or pressure overload or possibly concomitant to the valvular abnormalities, and, most importantly, to depict the crucial switch from adaptive (and reversible) to maladaptive (possibly irreversible) myocardial remodelling (*Graphical Abstract*). Cumulative evidence is already available on the use of advanced echocardiographic techniques and CMR unique capabilities to predict adverse myocardial remodelling and therefore refine timing for intervention. However, these imaging biomarkers are still largely underused, possibly because of lack of availability, standardization (including reproducibility, identification of cut-off values with clinical significance and comparison among different vendors) and prospective validation. The application in clinical practice particularly of novel CMR imaging biomarkers such as native T1 mapping, ECV, but also strain measures, will need to follow a careful implementation roadmap which has been outlined in a specific SCMR document.^17^ The access continues to improve as all CMR vendors already have T1 mapping sequences available and many commercial solutions for post-processing the data analysis. Nevertheless, the clinical implementation and expertise for the interpretation and reporting needs to be coupled with important review of the data acquisition, imaging quality, variability of the measures^64,90,102^ and attention to potential confounders (including loading conditions) or artefacts.^17^ Of note, since ECV calculation is derived from a ratio of myocardial/blood pool T1 value changes, ECV values are typically not influenced by the pulse sequence and/or magnetic field strength (which occurs for myocardial T1 values). As a consequence, native or post-contrast T1 mapping values have greater variability limiting their clinical applicability in multicentre studies.

Finally, the results of ongoing randomized trials including these novel imaging biomarkers as inclusion criteria, and future research, possibly applying new diagnostic algorithms from machine learning on big data, are highly awaited to help changing our perspective from a valve-centred to a myocardium-focused approach in managing patients with degenerative VHD.

## Data Availability

No new data were generated or analysed in support of this research.

## References

[1] Nkomo VT , Gardin JM, Skelton TN, Gottdiener JS, Scott CG, Enriquez-Sarano M. Burden of valvular heart diseases: a population based study. Lancet 2006;368:1005–1011. 16980116 10.1016/S0140-6736(06)69208-8

[2] Iung B , Delgado V, Rosenhek R, Price S, Prendergast B, Wendler O, et al ; EORP VHD II investigators. Contemporary presentation and management of valvular heart disease: the EURObservational research programme valvular heart disease II survey. Circulation 2019;140:1156–1169. 31510787 10.1161/CIRCULATIONAHA.119.041080

[3] Vahanian A , Beyersdorf F, Praz F, Milojevic M, Baldus S, Bauersachs J, et al 2021 ESC/EACTS guidelines for the management of valvular heart disease: developed by the task force for the management of valvular heart disease of the European society of cardiology (ESC) and the European association for cardio-thoracic surgery (EACTS). Eur Heart J 2022;43:561–632.34453165

[4] Otto CM , Nishimura RA, Bonow RO, Carabello BA, Erwin JP III, Gentile F, et al 2020 ACC/AHA guideline for the management of patients with valvular heart disease: A report of the American college of cardiology/American heart association joint committee on clinical practice guidelines. Circulation 2021;143:e72–e227. 33332150 10.1161/CIR.0000000000000923

[5] Kalam K , Otahal P, Marwick TH. Prognostic implications of global LV dysfunction: a systematic review and meta-analysis of global longitudinal strain and ejection fraction. Heart 2014;100:1673–1680. 24860005 10.1136/heartjnl-2014-305538

[6] Anyanwu AC , Adams DH. Etiologic classification of degenerative mitral valve disease: barlow’s disease and fibroelastic deficiency. Semin Thorac Cardiovasc Surg 2007;19:90–96. 17870001 10.1053/j.semtcvs.2007.04.002

[7] Yang L-T , Ahn SW, Li Z, Benfari G, Mankad R, Takeuchi M, et al Mitral valve prolapse patients with less than moderate mitral regurgitation exhibit early cardiac chamber remodeling. J Am Soc Echocardiogr 2020;33:815–825.e2. 32222479 10.1016/j.echo.2020.01.016 PMC8193998

[8] Grigioni F , Clavel MA, Vanoverschelde JL, Tribouilloy C, Pizarro R, Huebner M, et al The MIDA mortality risk score: development and external validation of a prognostic model for early and late death in degenerative mitral regurgitation. Eur Heart J 2018;39:1281–1291. 29020352 10.1093/eurheartj/ehx465

[9] van Wijngaarden AL , Mantegazza V, Hiemstra YL, Volpato V, van der Bijl P, Pepi M, et al Prognostic impact of extra-mitral valve cardiac involvement in patients with primary mitral regurgitation. JACC Cardiovasc Imaging 2022;15:961–970. 35033499 10.1016/j.jcmg.2021.11.009

[10] Marciniak A , Claus P, Sutherland GR, Marciniak M, Karu T, Baltabaeva A, et al Changes in systolic left ventricular function in isolated mitral regurgitation. A strain rate imaging study. Eur Heart J 2007;28:2627–2636. 17526904 10.1093/eurheartj/ehm072

[11] Hiemstra YL , Tomsic A, van Wijngaarden SE, Palmen M, Klautz RJM, Bax JJ, et al Prognostic value of global longitudinal strain and etiology after surgery for primary mitral regurgitation. JACC Cardiovasc Imaging 2020;13:577–585. 31202761 10.1016/j.jcmg.2019.03.024

[12] Mentias A , Naji P, Gillinov AM, Rodriguez LL, Reed G, Mihaljevic T, et al Strain echocardiography and functional capacity in asymptomatic primary mitral regurgitation with preserved ejection fraction. J Am Coll Cardiol 2016;68:1974–1986. 27591831 10.1016/j.jacc.2016.08.030

[13] Alashi A , Mentias A, Patel K, Gillinov AM, Sabik JF, Popović Z, et al Synergistic utility of brain natriuretic peptide and left ventricular global longitudinal strain in asymptomatic patients with significant primary mitral regurgitation and preserved systolic function undergoing mitral valve surgery. Circ Cardiovasc Imaging 2016;9:e004451. 27342145 10.1161/CIRCIMAGING.115.004451

[14] van Wijngaarden AL , de Riva M, Hiemstra YL, van der Bijl P, Fortuni F, Bax JJ, et al Parameters associated with ventricular arrhythmias in mitral valve prolapse with significant regurgitation. Heart 2021;107:411–418. 33004425 10.1136/heartjnl-2020-317451

[15] Stassen J , van Wijngaarden AL, Butcher SC, Palmen M, Herbots L, Bax JJ, et al Prognostic value of left atrial reservoir function in patients with severe primary mitral regurgitation undergoing mitral valve repair. Eur Heart J Cardiovasc Imaging 2022. 10.1093/ehjci/jeac058 PMC9762939 35301525

[16] Penicka M , Vecera J, Mirica DC, Kotrc M, Kockova R, Van Camp G. Prognostic implications of magnetic resonance-derived quantification in asymptomatic patients with organic mitral regurgitation: comparison with Doppler echocardiography-derived integrative approach. Circulation 2018;137:1349–1360. 29269390 10.1161/CIRCULATIONAHA.117.029332

[17] Messroghli DR , Moon JC, Ferreira VM, Grosse-Wortmann L, He T, Kellman P, et al Clinical recommendations for cardiovascular magnetic resonance mapping of T1, T2, T2* and extracellular volume: A consensus statement by the society for cardiovascular magnetic resonance (SCMR) endorsed by the European association for cardiovascular imaging (EACVI). J Cardiovasc Magn Reson 2017;19:75. 28992817 10.1186/s12968-017-0389-8 PMC5633041

[18] Kitkungvan D , Nabi F, Kim RJ, Bonow RO, Khan MA, Xu J, et al Myocardial fibrosis in patients with primary mitral regurgitation with and without prolapse. J Am Coll Cardiol 2018;72:823–834. 30115220 10.1016/j.jacc.2018.06.048

[19] Basso C , Perazzolo Marra M, Rizzo S, De Lazzari M, Giorgi B, Cipriani A, et al Arrhythmic mitral valve prolapse and sudden cardiac death. Circulation 2015;132:556–566. 26160859 10.1161/CIRCULATIONAHA.115.016291

[20] Constant Dit Beaufils AL , Huttin O, Jobbe-Duval A, Senage T, Filippetti L, Piriou N, et al Replacement myocardial fibrosis in patients with mitral valve prolapse: relation to mitral regurgitation, ventricular remodeling, and arrhythmia. Circulation 2021;143:1763–1774. 33706538 10.1161/CIRCULATIONAHA.120.050214

[21] de Meester de Ravenstein C , Bouzin C, Lazam S, Boulif J, Amzulescu M, Melchior J, et al Histological validation of measurement of diffuse interstitial myocardial fibrosis by myocardial extravascular volume fraction from modified Look-locker imaging (MOLLI) T1 mapping at 3 T. J Cardiovasc Magn Reson 2015;17:48. 26062931 10.1186/s12968-015-0150-0 PMC4464705

[22] Edwards NC , Moody WE, Yuan M, Weale P, Neal D, Townend JN, et al Quantification of left ventricular interstitial fibrosis in asymptomatic chronic primary degenerative mitral regurgitation. Circ Cardiovasc Imaging 2014;7:946–953. 25140067 10.1161/CIRCIMAGING.114.002397

[23] Kitkungvan D , Yang EY, El Tallawi KC, Nagueh SF, Nabi F, Khan MA, et al Extracellular volume in primary mitral regurgitation. JACC Cardiovasc Imaging 2021;14:1146–1160. 33341409 10.1016/j.jcmg.2020.10.010

[24] Kitkungvan D , Yang EY, El Tallawi KC, Nagueh SF, Nabi F, Khan MA, et al Prognostic implications of diffuse interstitial fibrosis in asymptomatic primary mitral regurgitation. Circulation 2019;140:2122–2124. 31841370 10.1161/CIRCULATIONAHA.119.043250 PMC7556709

[25] Pavon AG , Arangalage D, Pascale P, Hugelshofer S, Rutz T, Porretta AP, et al Myocardial extracellular volume by T1 mapping: a new marker of arrhythmia in mitral valve prolapse. J Cardiovasc Magn Reson 2021;23:102. 34517908 10.1186/s12968-021-00797-2 PMC8438990

[26] Liu B , Neil DAH, Bhabra M, Patel R, Barker TA, Nikolaidis N, et al Reverse myocardial remodeling following valve repair in patients with chronic severe primary degenerative mitral regurgitation. JACC Cardiovasc Imaging 2022;15:224–236. 34419393 10.1016/j.jcmg.2021.07.007

[27] Podlesnikar T , Delgado V, Bax JJ. Cardiovascular magnetic resonance imaging to assess myocardial fibrosis in valvular heart disease. Int J Cardiovasc Imaging 2018;34:97–112. 28642994 10.1007/s10554-017-1195-y PMC5797565

[28] Guglielmo M , Fusini L, Muscogiuri G, Baessato F, Loffreno A, Cavaliere A, et al T1 mapping and cardiac magnetic resonance feature tracking in mitral valve prolapse. Eur Radiol 2021;31:1100–1109. 32803414 10.1007/s00330-020-07140-w

[29] Lorell BH , Carabello BA. Left ventricular hypertrophy: pathogenesis, detection, and prognosis. Circulation 2000;102:470–479. 10908222 10.1161/01.cir.102.4.470

[30] Treibel TA , Lopez B, Gonzalez A, Menacho K, Schofield RS, Ravassa S, et al Reappraising myocardial fibrosis in severe aortic stenosis: an invasive and non-invasive study in 133 patients. Eur Heart J 2018;39:699–709. 29020257 10.1093/eurheartj/ehx353 PMC5888951

[31] Généreux P , Pibarot P, Redfors B, Mack MJ, Makkar RR, Jaber WA, et al Staging classification of aortic stenosis based on the extent of cardiac damage. Eur Heart J 2017;38:3351–3358. 29020232 10.1093/eurheartj/ehx381 PMC5837727

[32] Tastet L , Tribouilloy C, Maréchaux S, Vollema EM, Delgado V, Salaun E, et al Staging cardiac damage in patients with asymptomatic aortic valve stenosis. J Am Coll Cardiol 2019;74:550–563. 31345430 10.1016/j.jacc.2019.04.065

[33] Cavalcante JL , Rijal S, Abdelkarim I, Althouse AD, Sharbaugh MS, Fridman Y, et al Cardiac amyloidosis is prevalent in older patients with aortic stenosis and carries worse prognosis. J Cardiovasc Magn Reson 2017;19:98. 29212513 10.1186/s12968-017-0415-x PMC5719789

[34] Treibel TA , Fontana M, Gilbertson JA, Castelletti S, White SK, Scully PR, et al Occult transthyretin cardiac amyloid in severe calcific aortic stenosis: prevalence and prognosis in patients undergoing surgical aortic valve replacement. Circ Cardiovasc Imaging 2016;9:e005066. 27511979 10.1161/CIRCIMAGING.116.005066

[35] Scully PR , Treibel TA, Fontana M, Thornton GD, Hughes RK, Chadalavada S, et al Prevalence of cardiac amyloidosis in patients referred for transcatheter aortic valve replacement. J Am Coll Cardiol 2018;71:463–464. 29389364 10.1016/j.jacc.2017.11.037 PMC5780297

[36] Castano A , Narotsky DL, Hamid N, Khalique OK, Morgenstern R, DeLuca A, et al Unveiling transthyretin cardiac amyloidosis and its predictors among elderly patients with severe aortic stenosis undergoing transcatheter aortic valve replacement. Eur Heart J 2017;38:2879–2887. 29019612 10.1093/eurheartj/ehx350 PMC5837725

[37] Nitsche C , Scully PR, Patel KP, Kammerlander AA, Koschutnik M, Dona C, et al Prevalence and outcomes of concomitant aortic stenosis and cardiac amyloidosis. J Am Coll Cardiol 2021;77:128–139. 33181246 10.1016/j.jacc.2020.11.006 PMC7805267

[38] Vollema E , Sugimoto T, Shen M, Tastet L, Ng ACT, Abou R, et al Association of left ventricular global longitudinal strain with asymptomatic severe aortic stenosis: natural course and prognostic value. JAMA Cardiol 2018;3:839–847. 30140889 10.1001/jamacardio.2018.2288 PMC6233650

[39] Dahl JS , Magne J, Pellikka P, Donal E, Marwick TH. Assessment of subclinical left ventricular dysfunction in aortic stenosis. JACC Cardiovasc Imaging 2019;12:163–171. 30621988 10.1016/j.jcmg.2018.08.040

[40] Thellier AA , Appert L B, Leman B, Marsou W, et al Prognostic importance of left ventricular global longitudinal strain in patients with severe aortic stenosis and preserved ejection fraction. J Am Soc Echocardiogr 2020;33:1454–1464. 32919856 10.1016/j.echo.2020.07.002

[41] Magne J , Cosyns B, Popescu BA, Carstensen HG, Dahl J, Desai MY, et al Distribution and prognostic significance of left ventricular global longitudinal strain in asymptomatic significant aortic stenosis: an individual participant data meta-analysis. JACC Cardiovasc Imaging 2019;12:84–92. 30621997 10.1016/j.jcmg.2018.11.005

[42] Delgado V , Tops LF, van Bommel RJ, van der Kley F, Marsan NA, Klautz RJ, et al Strain analysis in patients with severe aortic stenosis and preserved left ventricular ejection fraction undergoing surgical valve replacement. Eur Heart J 2009;30:3037–3047. 19726436 10.1093/eurheartj/ehp351

[43] Fortuni F , Butcher SC, van der Kley F, Lustosa RP, Karalis I, de Weger A, et al Left ventricular myocardial work in patients with severe aortic stenosis. J Am Soc Echocardiogr 2021;34:257–266. 33181281 10.1016/j.echo.2020.10.014

[44] Carroll JD , Carroll EP, Feldman T, Ward DM, Lang RM, McGaughey D, et al Sex-associated differences in left ventricular function in aortic stenosis of the elderly. Circulation 1992;86:1099–1107. 1394918 10.1161/01.cir.86.4.1099

[45] Aurigemma GP , Gaasch WH. Gender differences in older patients with pressure-overload hypertrophy of the left ventricle. Cardiology 1995;86:310–317. 7553706 10.1159/000176895

[46] Dobson LE , Fairbairn TA, Musa TA, Uddin A, Mundie CA, Swoboda PP, et al Sex-related differences in left ventricular remodeling in severe aortic stenosis and reverse remodeling after aortic valve replacement: A cardiovascular magnetic resonance study. Am Heart J 2016;175:101–111. 27179729 10.1016/j.ahj.2016.02.010

[47] Treibel TA , Kozor R, Fontana M, Torlasco C, Reant P, Badiani S, et al Sex dimorphism in the myocardial response to aortic stenosis. JACC Cardiovasc Imaging 2018;11:962–973. 29153564 10.1016/j.jcmg.2017.08.025 PMC6278887

[48] Schwarz F , Flameng W, Schaper J, Langebartels F, Sesto M, Hehrlein F, et al Myocardial structure and function in patients with aortic valve disease and their relation to postoperative results. Am J Cardiol 1978;41:661–669. 645569 10.1016/0002-9149(78)90814-7

[49] Cheitlin MD , Cheitlin MD, Robinowitz M, Hoffman JI, Bharati S, Lev M. The distribution of fibrosis in the left ventricle in congenital aortic stenosis and coarctation of the aorta. Circulation 1980;62:823–830. 7408155 10.1161/01.cir.62.4.823

[50] Everett RJ , Tastet L, Clavel MA, Chin CWL, Capoulade R, Vassiliou VS, et al Progression of hypertrophy and myocardial fibrosis in aortic stenosis: A multicenter cardiac magnetic resonance study. Circ Cardiovasc Imaging 2018;11:e007451. 29914867 10.1161/CIRCIMAGING.117.007451 PMC6023592

[51] Debl K , Djavidani B, Buchner S, Lipke C, Nitz W, Feuerbach S, et al Delayed hyperenhancement in magnetic resonance imaging of left ventricular hypertrophy caused by aortic stenosis and hypertrophic cardiomyopathy: visualisation of focal fibrosis. Heart 2006;92:1447–1451. 16606864 10.1136/hrt.2005.079392 PMC1861032

[52] Barone-Rochette G , Pierard S, De Meester de Ravenstein C, Seldrum S, Melchior J, Maes F, et al Prognostic significance of LGE by CMR in aortic stenosis patients undergoing valve replacement. J Am Coll Cardiol 2014;64:144–154. 25011718 10.1016/j.jacc.2014.02.612

[53] Musa TA , Treibel TA, Vassiliou VS, Captur G, Singh A, Chin C, et al Myocardial scar and mortality in severe aortic stenosis. Circulation 2018;138:1935–1947. 30002099 10.1161/CIRCULATIONAHA.117.032839 PMC6221382

[54] Lee SP , Park SJ, Kim YJ, Chang SA, Park EA, Kim HK, et al Early detection of subclinical ventricular deterioration in aortic stenosis with cardiovascular magnetic resonance and echocardiography. J Cardiovasc Magn Reson 2013;15:72. 23984681 10.1186/1532-429X-15-72 PMC3766067

[55] Weidemann F , Herrmann S, Stork S, Niemann M, Frantz S, Lange V, et al Impact of myocardial fibrosis in patients with symptomatic severe aortic stenosis. Circulation 2009;120:577–584. 19652094 10.1161/CIRCULATIONAHA.108.847772

[56] Treibel TA , Kozor R, Schofield R, Benedetti G, Fontana M, Bhuva AN, et al Reverse myocardial remodeling following valve replacement in patients with aortic stenosis. J Am Coll Cardiol 2018;71:860–871. 29471937 10.1016/j.jacc.2017.12.035 PMC5821681

[57] Kim WK , Rolf A, Liebetrau C, Van Linden A, Blumenstein J, Kempfert J, et al Detection of myocardial injury by CMR after transcatheter aortic valve replacement. J Am Coll Cardiol 2014;64:349–357. 25060368 10.1016/j.jacc.2014.03.052

[58] Dobson LE , Musa TA, Uddin A, Fairbairn TA, Swoboda PP, Ripley DP, et al Post-procedural myocardial infarction following surgical aortic valve replacement and transcatheter aortic valve implantation. EuroIntervention 2017;13:e153–e160. 28117280 10.4244/EIJ-D-16-00558

[59] Bing R , Everett RJ, Tuck C, Semple S, Lewis S, Harkess R, et al Rationale and design of the randomized, controlled early valve replacement guided by biomarkers of left ventricular decompensation in asymptomatic patients with severe aortic stenosis (EVOLVED) trial. Am Heart J 2019;212:91–100. 30978556 10.1016/j.ahj.2019.02.018

[60] Schwarz F , Flameng W, Schaper J, Hehrlein F. Correlation between myocardial structure and diastolic properties of the heart in chronic aortic valve disease: effects of corrective surgery. Am J Cardiol 1978;42:895–903. 727140 10.1016/0002-9149(78)90673-2

[61] Milano AD , Faggian G, Dodonov M, Dodonov M, Golia G, Tomezzoli A, et al Prognostic value of myocardial fibrosis in patients with severe aortic valve stenosis. J Thorac Cardiovasc Surg 2012;144:830–837. 22244555 10.1016/j.jtcvs.2011.11.024

[62] Flett AS , Hayward MP, Ashworth MT, Hansen MS, Taylor AM, Elliott PM, et al Equilibrium contrast cardiovascular magnetic resonance for the measurement of diffuse myocardial fibrosis: preliminary validation in humans. Circulation 2010;122:138–144. 20585010 10.1161/CIRCULATIONAHA.109.930636

[63] Bull S , White SK, Piechnik SK, Flett AS, Ferreira VM, Loudon M, et al Human non-contrast T1 values and correlation with histology in diffuse fibrosis. Heart 2013;99:932–937. 23349348 10.1136/heartjnl-2012-303052 PMC3686317

[64] Everett RJ , Treibel TA, Fukui M, Lee H, Rigolli M, Singh A, et al Extracellular myocardial volume in patients with aortic stenosis. J Am Coll Cardiol 2020;75:304–316. 31976869 10.1016/j.jacc.2019.11.032 PMC6985897

[65] Rost C , Korder S, Wasmeier G, Wu M, Klinghammer L, Flachskampf FA, et al Sequential changes in myocardial function after valve replacement for aortic stenosis by speckle tracking echocardiography. Eur J Echocardiogr 2010;11:584–589. 20200001 10.1093/ejechocard/jeq017

[66] Carasso S , Cohen O, Mutlak D, Adler Z, Lessick J, Reisner SA, et al Differential effects of afterload on left ventricular long- and short-axis function: insights from a clinical model of patients with aortic valve stenosis undergoing aortic valve replacement. Am Heart J 2009;158:540–545. 19781412 10.1016/j.ahj.2009.07.008

[67] Lim E , Ali A, Theodorou P, Sousa I, Ashrafian H, Chamageorgakis T, et al Longitudinal study of the profile and predictors of left ventricular mass regression after stentless aortic valve replacement. Ann Thorac Surg 2008;85:2026–2029. 18498814 10.1016/j.athoracsur.2008.02.023

[68] Repossini A , Rambaldini M, Lucchetti V, Da Col U, Cesari F, Mignosa C, et al Early clinical and haemodynamic results after aortic valve replacement with the freedom SOLO bioprosthesis (experience of Italian multicenter study). Eur J Cardiothorac Surg 2012;41:1104–1110. 22315358 10.1093/ejcts/ezr140

[69] Beach JM , Mihaljevic T, Rajeswaran J, Marwick T, Edwards ST, Nowicki ER, et al Ventricular hypertrophy and left atrial dilatation persist and are associated with reduced survival after valve replacement for aortic stenosis. J Thorac Cardiovasc Surg 2014;147:362–369.e8. 23312984 10.1016/j.jtcvs.2012.12.016

[70] Gotzmann M , Lindstaedt M, Bojara W, Mugge A, Germing A. Hemodynamic results and changes in myocardial function after transcatheter aortic valve implantation. Am Heart J 2010;159:926–932. 20435207 10.1016/j.ahj.2010.02.030

[71] Lamb HJ , Beyerbacht HP, de Roos A, van der Laarse A, Vliegen HW, Leujes F, et al Left ventricular remodeling early after aortic valve replacement: differential effects on diastolic function in aortic valve stenosis and aortic regurgitation. J Am Coll Cardiol 2002;40:2182–2188. 12505232 10.1016/s0735-1097(02)02604-9

[72] Fairbairn TA , Steadman CD, Mather AN, Motwani M, Blackman DJ, Plein S, et al Assessment of valve haemodynamics, reverse ventricular remodelling and myocardial fibrosis following transcatheter aortic valve implantation compared to surgical aortic valve replacement: a cardiovascular magnetic resonance study. Heart 2013;99:1185–1191. 23749779 10.1136/heartjnl-2013-303927 PMC3747520

[73] La Manna A , Sanfilippo A, Capodanno D, Salemi A, Cadoni A, Cascone I, et al Left ventricular reverse remodeling after transcatheter aortic valve implantation: a cardiovascular magnetic resonance study. J Cardiovasc Magn Reson 2013;15:39. 23692630 10.1186/1532-429X-15-39 PMC3673841

[74] Dobson LE , Musa TA, Uddin A, Fairbairn TA, Swoboda PP, Erhayiem B, et al Acute reverse remodelling after transcatheter aortic valve implantation: A link between myocardial fibrosis and left ventricular mass regression. Can J Cardiol 2016;32:1411–1418. 27523272 10.1016/j.cjca.2016.04.009

[75] Flett AS , Sado DM, Quarta G, Mirabel M, Pellerin D, Herrey AS, et al Diffuse myocardial fibrosis in severe aortic stenosis: an equilibrium contrast cardiovascular magnetic resonance study. Eur Heart J Cardiovasc Imaging 2012;13:819–826. 22634740 10.1093/ehjci/jes102

[76] Fukui M , Annabi M, Rosa VEE, Ribeiro HB, Stanberry LI, Clavel MA, et al Comprehensive myocardial characterization using cardiac magnetic resonance associates with outcomes in low gradient severe aortic stenosis. Eur Heart J Cardiovasc Imaging 2022.. Online ahead of print. 10.1093/ehjci/jeac089 35613021

[77] Ternacle J , Krapf L, Mohty D, Magne J, Nguyen A, Galat A, et al Aortic stenosis and cardiac amyloidosis: JACC review topic of the week. J Am Coll Cardiol 2019;74:2638–2651. 31753206 10.1016/j.jacc.2019.09.056

[78] Taniguchi K , Kawamaoto T, Kuki S, Masai T, Mitsuno M, Nakano S, et al Left ventricular myocardial remodeling and contractile state in chronic aortic regurgitation. Clin Cardiol 2000;23:608–614. 10941548 10.1002/clc.4960230812 PMC6654784

[79] Lang RM , Badano LP, Mor-Avi V, Afilalo J, Armstrong A, Ernande L, et al Recommendations for cardiac chamber quantification by echocardiography in adults: an update from the American society of echocardiography and the European association of cardiovascular imaging. J Am Soc Echocardiog 2015;28:1–39.e14. 10.1016/j.echo.2014.10.003 25559473

[80] Anand V , Yang L, Luis SA, Padang R, Michelena HI, Tsay JL, et al Association of left ventricular volume in predicting clinical outcomes in patients with aortic regurgitation. J Am Soc Echocardiogr 2021;34:352–359. 33253815 10.1016/j.echo.2020.11.014

[81] Yang LT , Anand V, Zambito EI, Pellikka PA, Scott CG, Thapa P, et al Association of echocardiographic left ventricular End-systolic volume and volume-derived ejection fraction with outcome in asymptomatic chronic aortic regurgitation. JAMA Cardiol 2021;6:189–198. 33146680 10.1001/jamacardio.2020.5268 PMC7643045

[82] Cayli M , Kanadasi M, Akpinar O, Usal A, Poyrazoglu H. Diastolic function predicts outcome after aortic valve replacement in patients with chronic severe aortic regurgitation. Clin Cardiol 2009;32:E19–E23. 19455677 10.1002/clc.20437 PMC6653483

[83] de Meester C , Gerber BL, Vancraeynest D, Pouleur AC, Noirhomme P, Pasquet A, et al Do guideline-based indications result in an outcome penalty for patients with severe aortic regurgitation? JACC Cardiovasc Imaging 2019;12:2126–2138. 30660551 10.1016/j.jcmg.2018.11.022

[84] Yang LT , Michelena HI, Scott CG, Enriquez-Sarano M, Pislaru SV, Schaff HV, et al Outcomes in chronic hemodynamically significant aortic regurgitation and limitations of current guidelines. J Am Coll Cardiol 2019;73:1741–1752. 30846339 10.1016/j.jacc.2019.01.024

[85] Mentias A , Feng K, Alashi A, Rodriguez LL, Gillinov AM, Johnston DR, et al Long-Term outcomes in patients with aortic regurgitation and preserved left ventricular ejection fraction. J Am Coll Cardiol 2016;68:2144–2153. 27855803 10.1016/j.jacc.2016.08.045

[86] Ewe SH , Haeck ML, Ng AC, Witkowski TG, Auger D, Leong DP, et al Detection of subtle left ventricular systolic dysfunction in patients with significant aortic regurgitation and preserved left ventricular ejection fraction: speckle tracking echocardiographic analysis. Eur Heart J Cardiovasc Imaging 2015;16:992–999. 25733208 10.1093/ehjci/jev019

[87] Alashi A , Mentias A, Abdallah A, Feng K, Gillinov M, Rodriguez LL, et al Incremental prognostic utility of left ventricular global longitudinal strain in asymptomatic patients with significant chronic aortic regurgitation and preserved left ventricular ejection fraction. JACC Cardiovasc Imaging 2018;11:673–682. 28624411 10.1016/j.jcmg.2017.02.016

[88] Meucci MC , Butcher SC, Galloo X, van der Velde ET, Marsan NA, Bax JJ, et al Noninvasive left ventricular myocardial work in patients with chronic aortic regurgitation and preserved left ventricular ejection fraction. J Am Soc Echocardiogr 2022;35:703–711.e3. 35091069 10.1016/j.echo.2022.01.008

[89] Postigo A , Pérez-David E, Revilla A, Raquel LA, González-Mansilla A, Prieto-Arévalo R, et al A comparison of the clinical efficacy of echocardiography and magnetic resonance for chronic aortic regurgitation. Eur Heart J Cardiovasc Imaging 2022;23:392–401. 33332549 10.1093/ehjci/jeaa338

[90] Malahfji M , Senapati A, Tayal B, Nguyen DT, Graviss EA, Nagueh SF, et al Myocardial scar and mortality in chronic aortic regurgitation. J Am Heart Assoc 2020;9:e018731. 33241753 10.1161/JAHA.120.018731 PMC7763777

[91] Senapati A , Malahfji M, Debs D, Yang EY, Nguyen DT, Graviss EA, et al Regional replacement and diffuse interstitial fibrosis in aortic regurgitation: prognostic implications from cardiac magnetic resonance. JACC Cardiovasc Imaging 2021;14:2170–2182. 34274265 10.1016/j.jcmg.2021.04.028

[92] Fernández-Golfín C , Hinojar-Baydes R, González-Gómez A, Monteagudo JM, Esteban A, Alonso-Salinas G, et al Prognostic implications of cardiac magnetic resonance feature tracking derived multidirectional strain in patients with chronic aortic regurgitation. Eur Radiol 2021;31:5106–5115. 33449184 10.1007/s00330-020-07651-6

[93] Khalique OK , Cavalcante JL, Shah D, Guta AC, Zhan Y, Piazza N, et al Multimodality imaging of the tricuspid valve and right heart anatomy. JACC Cardiovasc Imaging 2019;12:516–531. 30846125 10.1016/j.jcmg.2019.01.006

[94] Chen CA , Dusenbery SM, Valente AM, Powell AJ, Geva T. Myocardial ECV fraction assessed by CMR is associated with type of hemodynamic load and arrhythmia in repaired tetralogy of fallot. JACC Cardiovasc Imaging 2016;9:1–10. 26684969 10.1016/j.jcmg.2015.09.011

[95] Haddad F , Doyle R, Murphy DJ, Hunt SA. Right ventricular function in cardiovascular disease, part II: pathophysiology, clinical importance, and management of right ventricular failure. Circulation 2008;117:1717–1731. 18378625 10.1161/CIRCULATIONAHA.107.653584

[96] Ebata R , Fujioka T, Diab SG, Pieles G, Ishii R, Ide H, et al Asymmetric regional work contributes to right ventricular fibrosis, inefficiency, and dysfunction in pulmonary hypertension versus regurgitation. J Am Soc Echocardiogr 2021;34:537–550.e3. 33383122 10.1016/j.echo.2020.12.011

[97] Gold J , Akazawa Y, Sun M, Hunter KS, Friedberg MK. Relation between right ventricular wall stress, fibrosis, and function in right ventricular pressure loading. Am J Physiol Heart Circ Physiol 2020;318:H366–H377. 31886720 10.1152/ajpheart.00343.2019

[98] Prihadi EA , van der Bijl P, Dietz M, Abou R, Vollema EM, Marsan NA, et al Prognostic implications of right ventricular free wall longitudinal strain in patients with significant functional tricuspid regurgitation. Circ Cardiovasc Imaging 2019;12:e008666. 30879327 10.1161/CIRCIMAGING.118.008666

[99] Fortuni F , Butcher SC, Dietz MF, van der Bijl P, Prihadi EA, De Ferrari GM, et al Right ventricular-pulmonary arterial coupling in secondary tricuspid regurgitation. Am J Cardiol 2021;148:138–145. 33667451 10.1016/j.amjcard.2021.02.037

[100] Sanz J , Sanchez-Quintana D, Bossone E, Bogaard HJ, Naeije R. Anatomy, function, and dysfunction of the right ventricle: JACC state-of-the-art review. J Am Coll Cardiol 2019;73:1463–1482. 30922478 10.1016/j.jacc.2018.12.076

[101] Lopes BBC , Hashimoto G, Bapat VN, Sorajja P, Scherer MD, Cavalcante JL. Cardiac computed tomography and magnetic resonance imaging of the tricuspid valve: preprocedural planning and postprocedural follow-up. Interv Cardiol Clin 2022;11:27–40. 34838295 10.1016/j.iccl.2021.09.004

[102] Kammerlander AA , Kraiger JA, Nitsche C, Donà C, Duca F, Zotter-Tufaro C, et al Global longitudinal strain by CMR feature tracking is associated with outcome in HFPEF. JACC Cardiovasc Imaging 2019;12:1585–1587. 31005535 10.1016/j.jcmg.2019.02.016

